# The effects of drug market regulation on pharmaceutical prices in Europe: overview and evidence from the market of ACE inhibitors

**DOI:** 10.1186/2191-1991-1-18

**Published:** 2011-11-21

**Authors:** Fritz von der Schulenburg, Sotiris Vandoros, Panos Kanavos

**Affiliations:** 1 LSE Health London School of Economics, Houghton Street London WC2A, UK; 2 University of Vienna Dr Karl Lueger Ring 1 Vienna 1010 Austria

**Keywords:** Regulation, Pharmaceuticals, Panel Data, Europe

## Abstract

This study provides an overview of policy measures targeting pharmaceutical expenditure in Europe and analyses their impact on originator pharmaceutical prices. Panel data methods are used to examine the market of ACE Inhibitors in six European countries (Denmark, France, Germany, Netherlands, Sweden, United Kingdom) over period 1991-2006. We find that although some measures are effective in reducing originator prices, others appear to have an insignificant effect. Results suggest that supply side measures such as mandatory generic substitution, regressive pharmacy mark-ups and claw-backs are effective in reducing pharmaceuticals prices. Results are not as strong for demand side measures. Profit controls and the use of cost-effectiveness analysis appear to have a negative effect on prices, while results on reference pricing are inconclusive. Findings also indicate that, although originator prices are not immediately affected by generic entry, they may be influenced by changes in generic prices post patent expiry.

## 1. Background

### 1.1 Introduction

The special nature of pharmaceutical markets (due to patent protection, third-party payers and low price elasticity) has led to the introduction of regulation in European markets. A variety of policy measures have been implemented in the European Union in order to control pharmaceutical prices. These measures differ significantly across countries, but have a common goal of efficient dispensing and keeping prices at reasonable levels, while elements of industrial policy can be found in some countries. Regulatory measures target both the demand side, as well as the supply side. However, although the aim of regulation is usually to decrease costs, it is not always the case that regulatory measures have the desired effect on prices and sales volume, because of market distortions.

Pharmaceutical market dynamics vary across Europe due to different regulatory frameworks. Empirical evidence has shown that heavily regulated markets with low prices tend to have fewer branded and generic launches than unregulated markets and demonstrate longer launch delays [1], while generic entry is less likely to occur in low-price economies and in countries with less regulation [2]. It has been shown that firms launch earlier in high-price EU countries [3]. There is evidence that firms launch strategically due to the direct influence of existing prices for the same drug in other countries. Empirical research comparing major EU countries with the US and Canada suggests that most European countries, which tend to be more regulated than the US, show a relatively higher presence of generic entrants [4].

The literature on the impact of generic entry on originator prices in Europe is inconclusive. However, empirical evidence from the US and other markets suggests that generic entry can lead to an increase in branded prices [5-8]. This phenomenon is known as the "generic paradox". The number of generic competitors might have an influence on the general price level via other factors [9,10]. However, it has been shown that originator prices do not increase post patent expiry [11]. Another study suggests that in the US, generic entry does not lead to a decrease in originator prices, but further price growth is "muted" [12]. In addition, the number of branded substitutes appears to have a negative effect on launch prices of new products [13,14]. There is evidence that generic prices decrease over time, which indicates the presence of generic competition among generic products in addition to some level of perceived product differentiation compared to branded pharmaceuticals. The market share of generics for certain products usually becomes quite large in short periods in most countries. After one year of entry, generic pharmaceuticals achieved a 44% market share in the US market [7,9].

With regards to reference pricing, it has been suggested that this policy has a marginally negative impact on prices [4]. Also, previous studies have analysed a change in Norway in 1993 from a price cap system to a reference based pricing system and its influence on pharmaceutical prices [15,16]. Findings showed that the reform led to lower originator and generic prices within the reference groups and an increase in generic market shares. However, it should be noted that these results do not necessarily imply that the decline would have been smaller if there had been no market intervention at all [17]. A study on Germany found that savings accumulated from implementing reference pricing were equal to nine percent of total drug expenditure [18]. Another policy intervention is tendering, which has been introduced in the market of some molecules in the Netherlands and Germany. Prices in the Netherlands decreased up to 92% as a result of tendering [19]. Evidence from Germany suggests that reference pricing and tendering have 'additive effects' [20]. Finally, an interesting study on supply-side measures has concluded that regulation may hurt competition between branded drugs [21].

In order to maximise the effectiveness of supply-side measures, appropriate demand-side measures are necessary [22,23], combined with other volume control measures [24,25]. Demand-side regulations and incentives can aim at physician prescribing behaviour or pharmacy dispensing patterns [17], which may include financial or non-financial incentives [26]. Empirical evidence has shown that regulatory policies that encourage or oblige pharmacists to substitute branded pharmaceuticals lead to a significant increase in the market share of the substitutes [27]. Other demand-side measures such as regressive pharmaceutical margins and policies targeting physicians' prescribing behaviour (e.g. budgets) have a positive impact on generic market shares [28]. Although results of another study were quite similar, a natural experiment showed that the number of hospital admissions and referrals increased significantly after Germany's introduction of pharmaceutical budgets in 1994 [29].

### 1.2. Drug Market Regulation in EU countries

In this paper we study six countries (UK, Germany, France, Denmark, Netherlands and Sweden) in order to gain insight into the effects of various regulatory measures on originator and generic prices. The selection of the countries is based on two primary criteria. We have selected large and widely referenced markets like Germany, France and the UK. In addition we have chosen countries with an interesting policy mix, such as the Netherlands, Sweden, Denmark. Germany (which is the third largest pharmaceutical market in the world) provides an interesting combination of free pricing for in-patent drugs and reference pricing for off-patent drugs. The United Kingdom is also a large market and is known for its unique indirect price regulation via rate-of-return regulation (profit controls) and for explicitly tackling the demand-side through a mix of regulatory measures such as claw-backs and incentives (e.g. prescribing guidelines, monitoring and budgets). France has the highest health care expenditure relative to the GDP in the EU and is the third largest drug producer in the world, accounting for 7% of the world's pharmaceutical output. In the Netherlands, the use of health technology assessment is used for new drugs, while reference pricing applies in off-patent markets (a relatively recent development is the introduction of tendering for the procurement of a limited number of out-patient drugs. The same applies in Germany). Physician and pharmacist incentives also play an important role in the Netherlands. Sweden was included because reforms that have taken place in this country since 2002 provide an interesting case for the empirical analysis. Several reforms have also been introduced in Denmark over the past decade. By including these six selected countries, the empirical analysis offers insight into different regulatory environments. Table 1 provides an overview of policy options in regulated pharmaceutical markets and Table 2 provides an overview of regulatory policies implemented in each of the study countries.

**Table 1:** Overview on regulation on the supply- and demand-side.

Supply-side	Demand-side
Price Controls	Physicians
Based on:	Clinical practice
Clinical performance	Prescription guidelines
Economic performance	Education
Cost of existing treatments	Information
Cost-plus calculations	Monitoring/audit
International prices	Prescription quotas
Controlled price update	Pharmaceutical budgeting
Free Pricing	Overall budgets
Expenditure Control	Patients
Discounts	Cost sharing
Rebates	Information
Pay-back	Education
Price-volume agreements	OTC spending
Price freeze/price cuts	
	Pharmacies
Industrial Regulation	Generic substitution
Profit controls/Rate of return	Monetary incentives
Tax benefits	Claw-backs
	Margins
	Discounts

**Table 2:** Pharmaceutical Policies in six European countries.

	Sweden	Netherlands	Denmark	France	UK	Germany
**Reference Pricing**	X (since 1993)	X	X (since 1993)	X (since 2003)		X
**Substitution Mandatory**	X (since 2002)		X (since 1997)			X (since 2004)
**Generics Price Control**				X	X (since 2000)	
**Mark-up Regression**	X		X (until 2007)	X (with a pause between 1999-2003)	X	X (since 2003)
**Profit Control**					X	
**Clawback**		X (since 1998)			X (since 1997)	X (since 2004)
**Tax Funded Health Care System**	X		X		X	
**Cost-Efficiency Analysis**	X (since 2002)	X (since 1998)	X (since 2005, but not compulsory)		X (since 2000)	

#### Supply-side measures

A popular pricing policy, which is extensively used in Europe (Germany, Denmark, France, Sweden, Netherlands), is reference pricing. The reference price defines a reimbursement rate or level for all products within a specific group of drugs. The reference price reflects what is reimbursed by health insurance. If a product's price exceeds the reference price, the product may not be covered, or the patient may have to pay the difference out-of-pocket. The reference price may be set at the molecule level (thus applying to off-patent markets) or the therapeutic class level, in which case prices of in-patent drugs in the same class may also be subject to the reference price.

The use of evaluation methods to support reimbursement decisions is becoming increasingly popular in the EU. The use of cost-effectiveness analysis (and health technology assessment in general) influences the suppliers' pricing decisions, as setting a relatively high price (compared to therapeutic value) may have a negative impact on the decision of whether the product will be reimbursed or not. Cost-effectiveness analysis is a valuable tool in deciding upon whether to have a product reimbursed or not. Another way to regulate prices is through price controls. The launch price might depend on clinical performance, economic evaluation, costs of already existing similar treatments, the basis of calculation (e.g., average, lowest price), costs plus a certain profit margin or on international and national prices of the same product [30]. In some cases drugs can be freely priced.

Instead of directly interfering in pricing strategies of companies, rate-of-return regulation can mark a profit limit (as in the UK). This type of regulation is implemented in order to achieve reasonable prices while safeguarding the profitability of the innovative pharmaceutical industry. Another possibility is to give tax benefits for investments in R&D or in manufacturing capacity [30].

Various types of volume controls are also used in the EU. Such controls are used because price controls are often ineffective in controlling pharmaceutical expenditure due to rising utilization. Price-volume agreements can be reached through negotiations between the industry and authorities. Producers that exceed the agreed volume are penalized and obliged to decrease the price or pay back a certain amount or return a certain amount of their revenue to the purchaser. Paybacks are often used as thread methods for price-volume agreements. Price cuts also apply across the EU. Finally, rebates include any returns on sales made by a manufacturer to an institutional player [30].

#### Demand-side measures

Drug consumption can be influenced and regulated through different monetary incentives, regulation, schooling and exchange of information. Physicians act as patients' agents and express proxy demand, as they have more information about appropriate treatments than patients. Financial or non-financial incentives can be used to help physicians prescribe in a cost-effective way.

Efficient prescribing and the minimisation of risk for patients can be supported and upgraded by educational barriers (classification for physicians) and information methods. Computerized decision support, online prescribing advice and prescribing monitoring can help achieve improved prescribing patterns. Another way of influencing prescribing is through the implementation of prescription quotas and pharmaceutical budgets. These motivate physicians to be cost-conscious when it comes to selecting between alternative treatments. Prescribing by INN rather than by brand name and dispensing policies at the pharmacy level can also encourage efficient use of medicines. Generic substitution at the pharmacy encourages or obliges physicians to dispense generics instead of the corresponding originators. This helps increase generic market shares, cost-effectiveness and encourage generic entry. Furthermore, claw-backs are used by authorities to gain back part of the discounts that pharmacists receive on generic products.

Healthcare authorities also implement monetary incentives for pharmacists through mark-up schemes. Carefully designed regressive pharmacy margins make dispensing cheaper products more profitable for pharmacists, hence encouraging them to dispense generics rather than originators. A flat fee per prescription would normally not give any incentive to dispense cheaper or more expensive products. However, as pharmacists often receive discounts on generic products, a flat fee would probably also make them prefer generics to originators.

Patients also play a role in the pharmaceutical market, although this is limited because physicians usually express demand for drugs on their behalf. Due to high reimbursement levels in most EU countries, cost-consciousness is often low. Patient behaviour can be influenced by fees and cost sharing. Cost-sharing, which is the most common way of affecting patients, is used in many countries in different ways. Cost sharing might, for example, be set as a fixed co-payment for drugs (per item, per packet etc.) or a fixed fee paid to pharmacists. These payments may also be a variable percentage of the prescribed drug's price. Another possibility of affecting patients' behaviour lies in informational and educational campaigns. This might increase their awareness regarding differential co-payments, generic bioequivalence and rational use of pharmaceuticals.

Using an empirical investigation, the paper studies the impact that regulation has on pharmaceutical prices, and the effect of generic competition on market dynamics in six pharmaceutical markets in Europe (UK, Germany, the Netherlands, France, Denmark, Sweden) post patent expiry. The paper is organised as follows: Section 2 explains the methodology employed; section 3 provides the empirical results; section 4 provides a discussion and policy implications; section 5 concludes.

## 2. Methods

After having explained the various regulatory regimes in the six European countries considered in this study, we proceed to study the actual effects of the discussed regulatory measures on drug prices.

Pharmaceutical prices depend on patent protection, market structure and regulation. The presence of patent protection defines the market as a monopoly for a particular molecule. Post-patent expiry generics competitors are present, so the market of the particular molecule is no longer a monopoly, indicating that markets change significantly over a product's life cycle. Regulatory measures are implemented by authorities in order to prevent pharmaceutical prices from being very high and to enable access to medicines for eligible patients. Such measures heavily affect prices and market shares, so prices are expected to evolve differently depending on the level and the nature of regulation. The empirical model that we estimate in this section is based on these market dynamics.

According to economic theory and the nature of policies and other aspects of the pharmaceutical market, we have expectations with regards to the direction of their impact on prices. The number of competitors in the market is expected to have a negative impact on prices. The same applies to the presence of generic competitors, as generic entry means that competition in the market of the particular molecule is now present. Generic prices are expected to be positively associated with originator prices, as generics and originators of the same molecule are direct substitutes.

The goal of reference pricing is to keep prices at moderate levels, as in the presence of this policy the cheapest product gets reimbursed, creating an incentive for producers to reduce their price. Therefore, we expect this policy measure to have a negative effect on prices. Product substitution at the pharmacy and regressive mark-ups would normally also be expected to create downward pressure on prices, as the manufacturer of the product would decrease the price in order to have the product dispensed: A lower price could prevent generic substitution, while regressive mark-ups would often make pharmacists dispense cheaper products, therefore the manufacturer would decrease the price in order to have their product dispensed. Profit controls are not necessarily expected to decrease prices, as their goal is not only to provide affordable medicines, but also to ensure the presence of a viable pharmaceutical industry.

Data on ACE Inhibitors are used for the empirical analysis. The reason these drugs are used is because they belong to a high-volume class for a common disease (cardiovascular disease). Further, off-patent ACE Inhibitors face extensive generic competition due to the relatively high volume of ACE Inhibitor sales in the EU. Data on ACE Inhibitor prices were obtained from the IMS Midas database. These include actual public prices, deflated and converted to Euros. Data are reported quarterly, from 1991 to 2006. The sample includes all available ACE Inhibitors (captopril, enalapril, lisinopril, quinapril, ramipril, trandalopril, periodinopril, moexipril, fisinopril, benazepril, cilazapril, zofenopril, imidrapril, apriapril). Six countries were considered for the purpose of the analysis (Denmark, France, Germany, Netherlands, Sweden and the United Kingdom). Including different countries in the sample allows us to control for different regulatory regimes across markets and unobserved heterogeneity.

We create models in order to empirically test the impact of regulation on originator and generic prices (Equations (1), (2), (3) and (4)). These show the impact of market structure and policies on originator prices. The coefficients reveal whether each of the variables has a positive or negative impact on originator prices and whether their effect is statistically significant or not.

(1) $$\begin{array}{ccc} price_{it} & =\alpha +\beta_{0}+\beta_{1}generics_{it}+\beta_{2}rp_{it}+\beta_{3}sm_{it}+\beta_{4}mark_{it}+\beta_{5}profitc_{it} & \\ & +\beta_{6}clawback_{it}+\beta_{7}tax_{it}+\beta_{8}cea_{it}+\varepsilon_{it} \end{array}$$

(2) $$\begin{array}{ccc} price_{it} & =\alpha +\beta_{0}+\beta_{1}n_{it}+\beta_{2}rp_{it}+\beta_{3}sm_{it}+\beta_{4}mark_{it}+\beta_{5}profitc_{it} & \\ & +\beta_{6}clawback_{it}+\beta_{7}tax_{it}+\beta_{8}cea_{it}+v_{it} \end{array}$$

(3) $$\begin{array}{ccc} price_{it} & =\alpha +\beta_{0}+\beta_{1}genprice_{it}+\beta_{2}rp_{it}+\beta_{3}sm_{it}+\beta_{4}mark_{it} & \\ & +\beta_{5}profitc_{it}+\beta_{6}clawback_{it}+\beta_{7}tax_{it}+\beta_{8}cea_{it}+u_{it} \end{array}$$

(4) $$\begin{array}{ccc} price_{it} & =\alpha +\beta_{0}+\beta_{1}genprice_{it}+\beta_{2}n_{it}+\beta_{3}rp_{it}+\beta_{4}sm_{it}+\beta_{5}mark_{it} & \\ & +\beta_{6}profitc_{it}+\beta_{7}clawback_{it}+\beta_{8}tax_{it}+\beta_{9}cea_{it}+\mu_{it} \end{array}$$

*price *is the price of the originator product, measured in logs. *generics *is a dummy variable. It is 1 if generic competitors are present and zero otherwise. *genprice *is the price of the generic product. *n *indicates the number of generic competitors in the market of a particular molecule. It is zero when there are no generics in the market. *rf *is a policy dummy variable indicating the presence of reference pricing. *sm *is also a dummy variable indicating the presence or not of mandatory generic substitution at the pharmacy level. *mark *indicates the presence of regressive mark-ups for pharmacist remuneration. *profitc *is a dummy variable which takes the value of 1 if there are profit controls in place, and zero if there is no such measure present. *clawback *indicates the presence of claw-backs. *tax *is a dummy variable which shows whether the health system is tax-funded or not. *cea *indicates the explicit use of cost effectiveness analysis in decisions concerning drug reimbursement. Variables representing expenditure control methods and reimbursement rates were not included in the empirical model due to their different qualitative nature across countries, and price controls were excluded because of multicollinearity problems. The error terms in all four models are normally distributed, their mean is zero and variance is finite. Time dummies were also used to control for changes over time. Summary statistics are in Table 3.

**Table 3:** Summary Statistics.

Variable	**Obs**.	Mean	**S.E**.
*price*	3402	-2.410	0.988
*generics*	3998	0.425	0.494
*genprice*	1699	-2.983	1.164
*n*	3998	4.574	8.828
*rf*	3998	0.609	0.488
*sm*	3998	0.151	0.358
*mark*	3998	0.527	0.499
*profitc*	3998	0.168	0.374
*clawback*	3998	0.222	0.416
*tax*	3998	0.451	0.498
*cea*	3998	0.190	0.393
*contgen*	3998	0.398	0.490

Models (1) and (2) show the determinants of originator drug prices over the life cycle of a drug. They include observations before and after generic entry. Models (3) and (4) show the determinants of originator drug prices post patent expiry. The reason for this discrimination is that if generic prices are used, they restrict the model only to post patent expiry observations. Therefore, Models (3) and (4) only include these observations. Models (1) and (2) do not include generic prices as explanatory variables, but include all time periods available. Model (1) uses the presence of generics as a proxy for the effect of generic competition on originator prices, while models (2) and (4) use the number of generic entrants (*n*) to capture this effect.

Panel data estimation is used to estimate the two models. The panel identifier is determined at the molecule per country level. There are possible endogeneity problems in the estimation of Model (2). It is possible that not only do generic prices affect originator prices, but also originator prices affect generic prices. Therefore, we use instrumental variable estimation methods to overcome this problem. *contgen *(the presence of generic price controls) is used as an instrument because generic price controls affect the endogenous variable (generic prices), but do not directly affect originator prices.

## 3. Results

Estimation results of Models (1) and (2) are in Table 4 both for fixed effects and random effects. The Hausman test suggests that it is safe to use random effects, which offer a more efficient estimator. However, we report results for both fixed effects and random effects.

**Table 4:** Panel data estimation all markets.

Dependent variable: *price*				
	Model (1)		Model (2)	
	FE	RE	FE	RE
*generics*	0.014	0.011		
	(0.016)	(0.016)		
*N*			-0.006***	-0.007***
			(0.002)	(0.002)
*Rf*	0.086***	0.085***	0.089***	0.088***
	(0.019)	(0.019)	(0.019)	(0.019)
*Sm*	-0.248***	-0.247***	-0.240***	-0.239***
	(0.019)	(0.019)	(0.019)	(0.019)
*Mark*	-0.284***	-0.283***	-0.262***	-0.259***
	(0.031)	(0.031)	(0.031)	(0.031)
*profitc*		-0.281		-0.273
		(0.312)		(0.328)
*clawback*	-0.044**	-0.045**	-0.045**	-0.046**
	(0.020)	(0.020)	(0.020)	(0.020)
*Tax*		0.121		0.087
		(0.233)		(0.245)
*Cea*	-0.050**	-0.049**	-0.046**	-0.046**
	(0.021)	(0.021)	(0.021)	(0.021)
*constant*	-1.856***	-1.883***	-1.846***	-1.866***
	(0.042)	(0.142)	(0.042)	(0.149)

Observations	3402	3402	3402	3402
Rsq within	0.475	0.475	0.478	0.478
Rsq between	0.002	0.002	0.028	0.049
Rsq overall	0.036	0.038	0.077	0.086
Wald Chi-sq		2943.53		2989.86
F - statistic	44.88		45.37	

*, **, *** refer to statistical significance at the α = 1%, 5% and 10% respectively.

Standard errors in parenthesis.

Generic entry (captured by explanatory variable *generics*) has a statistically insignificant coefficient in Model (1), in both the fixed effects and the random effects approach, suggesting that generic competition does not lead to a decrease in originator prices. *sm*, *mark*, *clawback *and *cea *have a negative and statistically significant coefficient in both the random effects and fixed effects approach. These results show that mandatory generic substitution, regressive pharmacy mark-ups, claw-backs and the use of cost effectiveness analysis lead to lower originator prices. However, reference pricing appears to have a positive effect on originator prices. *profitc *and *tax *have a statistically insignificant coefficient, which means that profit controls do not affect originator prices and that the nature of the funding of health services does not influence prices.

In Model (2), the number of generic competitors is negative and statistically significant in both the fixed effects and random effects approach, meaning that an increase in the number of generic competitors leads to a decrease in originator prices. As in model (1), mandatory generic substitution, regressive mark-ups, claw-backs and the use of cost effectiveness analysis have a negative and statistically significant effect on originator prices in both the fixed effects and random effects approach. Again, reference pricing appears to have a positive effect on originator prices. Profit controls and the nature of the funding of health services again do not have a significant effect on prices.

Estimation results of Models (3) and (4), which consider off-patent markets only, are in Table 5. Results are similar to those of Models (1) and (2). However, results of Model (4) suggest the number of generic competitors does not influence on originator prices. Generic prices are positively correlated with originator prices, which is what is generally expected for substitutes. Reference pricing, mandatory generic substitution and regressive pharmacist mark-ups have a negative and statistically significant effect on originator prices, both in Model (3) and (4), for both fixed effects and random effects. Profit controls appear to affect originator prices negatively. Also, health systems that are tax-funded seem to demonstrate higher originator prices. Finally, the use of cost effectiveness analysis does not appear to have a significant effect on originator prices.

**Table 5:** Instrumental variable panel data estimation off-patent markets.

Dependent variable: *price*				
	Model (3)		Model (4)	
	FE	RE	FE	RE
*genprice*	0.524***	0.657***	0.523***	0.644***
	(0.111)	(0.147)	(0.114)	(0.134)
*N*			0.001	-0.002
			(0.003)	(0.003)
*Rf*	-0.090*	-0.121**	-0.089*	-0.118**
	(0.051)	(0.061)	(0.052)	(0.058)
*Sm*	-0.342***	-0.309***	-0.341***	-0.310***
	(0.034)	(0.039)	(0.035)	(0.037)
*Mark*	-0.303***	-0.292***	-0.302***	-0.282***
	(0.050)	(0.049)	(0.055)	(0.051)
*profitc*		-0.560***		-0.563***
		(0.189)		(0.199)
*clawback*	-0.058	-0.067*	-0.058	-0.067*
	(0.036)	(0.036)	(0.037)	(0.036)
*Tax*		0.377***		0.361***
		(0.114)		(0.126)
*Cea*	-0.047	-0.056	-0.045	-0.054
	(0.036)	(0.038)	(0.036)	(0.038)
*constant*	-0.889***	-0.683*	-0.754**	-0.546*
	(0.330)	(0.378)	(0.297)	(0.307)

Observations	1645	1645	1645	1645
Rsq within	0.511	0.490	0.511	0.493
Rsq between	0.796	0.906	0.796	0.903
Rsq overall	0.723	0.818	0.724	0.816
Wald chi sq	173361.16	1411.47	173341.90	1439.64

*, **, *** refer to statistical significance at the α = 1%, 5% and 10% respectively.

Standard errors in parenthesis.

The results of the econometric analysis provide insight into the determinants of originator drug prices. There is some weak evidence that the number of generic competitors leads to a decrease in originator prices, as a result of competition, which is what economic theory would predict. However, this seems to occur gradually, and generic entry does not have an immediate effect on prices. Mandatory generic substitution and regressive pharmacist mark-ups have a strong negative effect on originator prices, indicating the competition effect of generics on originators, as the originator price may adjust to competition in order to keep part of the market. These two measures appear to be the most effective with regards to originator prices. Some evidence also exists that profit controls, claw-backs and the explicit use of cost effectiveness analysis reduce originator prices. Profit controls are expected to prevent drug prices from becoming unreasonably high, and evidence on this policy appears to somehow follow this pattern. The use of cost-effectiveness analysis also seems to make producers price their products at reasonable levels, in order to avoid having them excluded from reimbursement. The evidence on reference pricing is inconclusive, as the direction of the impact of reference pricing on prices changes depending on the specification of the model. Cost containment, which is the goal of reference pricing, does not necessarily take place. Finally, generic prices are positively associated with originator prices.

## 4. Discussion

We have studied the impact of different policy measures that apply in various countries in different time periods on originator prices. We found strong empirical evidence that generic substitution and regressive pharmacy mark-ups have a negative effect on originator drug prices. Generic substitution enhances price competition, as more expensive products are substituted by cheaper alternatives at the pharmacy. This gives producers an incentive to reduce prices in order to have their products reimbursed by health insurance. Regressive pharmacy mark-ups have a similar effect. When pharmacists are penalised for dispensing more expensive products, there is an incentive for them to dispense cheaper alternatives. Therefore, by reducing the price, manufactures can make their product more likely to be dispensed. However, other policy measures do not appear to be as effective. Evidence on the impact of reference pricing, profit controls and the use of cost effectiveness analysis is less clear because the statistical significance of the results changes across model specifications. Generic entry does not seem to directly influence originator prices, but there may be a gradual effect through an increase in the number of generic competitors.

There have been concerns that originator products do not respond to generic competition or price regulation post patent expiry. The reason for such concern is that originator producers lost interest in a market after generic entry and did not try to keep a large market share by lowering prices. However, this analysis has showed that in the case of ACE Inhibitors, originator prices may indeed decrease as a result of generic competition and generic policies such as reference pricing, substitution and regressive mark-ups. Therefore, there is evidence that originator prices may not be completely irresponsive to competition.

Policy makers should encourage swift generic uptake, because this leads to direct savings, as generics are cheaper. However, generic prices must be significantly lower than originator prices because there are no R&D costs involved. Generic substitution and regressive pharmacy mark-ups can lead to savings and have a positive effect on competition. Other policies may have to be reviewed, as their impact may not be as strong as expected. For example, reference pricing may initially lead to price reductions, but may also discourage price competition in the long run. Producers may price their products at the reference price level, while having no incentive to make any further price reductions.

In any case, policy measures must be designed to fit the particular needs of each market, rather than simply copied from other countries. Promoting competition should be one of the authorities' primary goals, and policies should be implemented in areas that inefficiencies occur due to the special nature of pharmaceutical markets. Regulating prices is often necessary in order to keep prices at reasonable levels and safeguard access to care for patients, but the impact on future R&D should also not be ignored. As previous research has indicated, the introduction of new regulatory measures can lead to a slowdown in R&D [31].

## 5. Conclusions

This study has provided an overview of regulatory measures implemented in the European Union targeting drug prices. Findings suggest that supply side measures are effective in reducing pharmaceuticals prices. Mandatory generic substitution, regressive pharmacy mark-ups and claw-backs contribute to lower pharmaceutical prices. Findings on demand-side pricing policies are less clear. In most cases, profit controls and the use of cost effectiveness analysis appear to have a negative effect on prices, while results on the impact of reference pricing are inconclusive. In addition, although generic entry does not have an immediate effect on originator prices, the latter are subsequently influenced by changes in generic prices. This study is not without limitations. Our findings are relevant for the market of ACE Inhibitors and do not necessarily apply to any drug market. Future research can include a wider range of products from different therapeutic classes, in order to provide results that can be generalised and establish a link between regulation and prices. Finally, due to data availability and the selection of products and time series, it was not possible to observe the effect of tendering on pharmaceutical markets in Germany and the Netherlands, which is also something that future research could focus on.

## Competing interests

The authors declare that they have no competing interests.

## Authors' contributions

Study conception and design: FS, SV, PK. Policy background: FS. Data extraction: PK. Data requirements: FS. Econometric analysis: SV. Discussion and Conclusions: FS, SV, PK. Drafting of manuscript: FS, SV, PK.
